# Triple Valvulopathy and Jaccoud's Arthropathy: A Case Report and Literature Review

**DOI:** 10.1155/2016/4147068

**Published:** 2016-06-28

**Authors:** Ali Naderi Mahabadi, Bassam Alhaddad, Stanley Ballou

**Affiliations:** ^1^Department of Medicine, MetroHealth Medical Center, 2500 MetroHealth Drive, Cleveland, OH 44109, USA; ^2^Departments of Medicine and Rheumatology, MetroHealth Medical Center, 2500 MetroHealth Drive, Cleveland, OH 44109, USA

## Abstract

Cardiac involvement is fairly common in patients with systemic lupus erythematosus (SLE). It may involve all layers of the heart and coronary arteries as well as the heart valves. We report an extremely rare presentation of valvulitis and valvular dysfunction associated with systemic lupus erythematosus. This is the first case of lupus valvulitis which required three mechanical prosthetic valve replacements with disease recurrence leading to a fatal outcome. This is, in our point of view, the consequence of aggressive natural history of the disease and perhaps late diagnosis and treatment of underlying SLE which was unsuccessful.

## 1. Introduction

Cardiac involvement is fairly common in patients with systemic lupus erythematosus (SLE). All layers of the myocardium and coronary arteries as well as heart valves may be involved. Valvular disease in SLE has been described in up to 27% of patients [1]. The valvular involvement ranges from small nodules to large lesions known as Libman-Sacks endocarditis, causing regurgitant lesions as well as stenotic disease. Mitral valve regurgitation, in particular, is the most common valvular abnormality (26%), compared to aortic and tricuspid valve (7% each) [2]. Hemodynamically significant disease requiring valve repair or replacement is uncommon. Here, we report a 33-year-old patient with SLE who required mitral, aortic, and tricuspid valve replacement secondary to lupus valvulitis.

## 2. Case

A 33-year-old African American female with no past medical history presented to the emergency department with a two-week history of progressive shortness of breath, chest pain on exertion, bilateral pitting edema of the legs, and ascites. An echocardiogram showed severe aortic stenosis, moderate mixed mitral valve disease, and severe tricuspid regurgitation with preserved left ventricular ejection fraction. A right and left heart catheterization showed moderate-to-severe pulmonary hypertension with unobstructed coronary arteries. She did not have any known history of rheumatic fever. She had clinical appearance of Jaccoud's arthropathy of hands with reversible hyperflexion of all proximal interphalangeal joints and hyperextension of distal interphalangeal joints. She reported 2 years of hand pain and was told that she had Rheumatoid Arthritis (RA) at an express care clinic but never obtained further medical care for her hands. Further workup showed negative serologies for RA, hepatitis C virus, and hepatitis B virus; positive tests for anti-nuclear, anti-dsDNA, anti-Smith, and anti-histone antibodies; and low titer beta-2 glycoprotein with negative lupus anticoagulant and cardiolipin antibodies. Her C3 level was low at 26 mg/dL and C4 was undetectable. A urine protein-to-creatinine ratio was 1000 mg/g.

She was diagnosed with systemic lupus erythematosus with cardiac and renal involvement and was started on mycophenolate 1000 mg twice daily and 20 mg prednisone, with resolution of proteinuria. A renal biopsy was not performed during this time as the need for cardiac surgery was deemed urgently necessary. One month later, she underwent triple mechanical prosthetic valve replacement surgery and pathological examination confirmed chronic valvulitis. Postoperatively, she was weaned off prednisone and continued on mycophenolate. Unfortunately, the patient was readmitted to another institution several months later with worsening heart failure. Transesophageal echocardiogram confirmed presence of vegetation on all mechanical valves with evidence of valve dysfunction. Patient underwent surgical intervention to replace all 3 valves which was unfortunately complicated by a massive stroke and subsequently patient died. Postmortem cultures and tissue histology did not reveal any organisms confirming sterile recurrent endocarditis.

## 3. Discussion

From our literature review and to our knowledge, this is the first case of lupus valvulitis requiring three mechanical prosthetic valve replacements with disease recurrence leading to a fatal outcome. Another interesting aspect of our case is the absence of antiphospholipid syndrome, which is strongly associated with lupus valvulopathy [3]. An association between hypocomplementemia, Jaccoud's arthropathy, and valvulopathy, however, has been described in case reports and a recent retrospective study [4].

No studies have been done to address the question of the optimal surgical intervention for valve damage in SLE. In general, valve repair is preferred over replacement for mitral diseases in eligible patients [5]. However, in SLE valvulitis, older studies suggest that results of mitral valve replacement are usually superior to repair in providing better outcomes [6] due to high rate of valve failure requiring subsequent replacement. A recent review article suggested that mitral valve repair should still be considered in specific patients (controlled SLE, young age, and localized damage with otherwise normal leaflets) [7]. Based on the available literature, there is no consensus with regard to management of valve damage from SLE endocarditis (repair, mechanical or bioprosthetic valve replacement). Macroscopic examination of the valve during the surgery and the degree of valvular calcification and fibrosis are the most important factors in making treatment decisions. Other factors include SLE disease activity, age, and consideration for lifelong anticoagulation.

Prosthetic valve dysfunction in SLE has been described in a few case reports over varying time intervals, with a high mortality and morbidity rate. In some patients, hemodynamically significant valvular dysfunction can be controlled with conservative management such as immunosuppression, anticoagulation, and evidence-based heart failure treatment [8]. However, if severe symptomatic valvular dysfunction persists, valve surgery may be again required [9].

Our case is an extreme case of valvulitis and valvular dysfunction associated with systemic lupus erythematosus, which in our point of view was the consequence of the aggressive natural history of the disease and perhaps also late diagnosis and treatment of her underlying SLE.
